# Supplementary material for: Counselling experiences among men having sex with men and living with HIV in Malaysia
Source: PLoS One. 2022 Sep 15;17(9):e0274251. doi: 10.1371/journal.pone.0274251 (PMC9477305; doi:10.1371/journal.pone.0274251)
Supplement: S1 Data — (DOCX) [file pone.0274251.s001.docx]

**Data 1 - Excerpts of participants’ stories based on themes**

|  | | | | |
| --- | --- | --- | --- | --- |
| **Participant** | **Feeling of emptiness** | | **Feeling of hopelessness** | |
|  | **Uncomfortable** | **Demotivated** | **No hope** | **No trust** |
| Participant 1 | I was asked to attend the session every time I attended the treatment. Honestly, I was not comfortable. That does not mean counselling is not good, but the feeling remained after I left the counselling session. For me, it was nothing and useless because I know there will be no change with HIV. | I have no interest in doing anything I used to. Especially in terms of my career, most days I feel unmotivated to continue working. However, I have to pretend like nothing has happened to me. But it is not easy. | What can I hope for when living with this disease? There is nothing for me. I have started to feel like everything is wrong for me. | I admit that I am not the type of person who easily shares anything about myself. In the counselling sessions, I noticed and could feel that the counsellor was trying to get some information about me, for example, my personal issues. For me, it is not easy to share and to make people understand it. My situation will never get better. No one can help me. So, I only chose to discuss treatments. |
| Participant 2 | I don’t think that it would be easy for me to get used to a service like counselling, even though I know they just want to help me. But I am blank with this disease. I had never expected HIV in my body and now my life will end with this. | When I was diagnosed with HIV, the obvious thing I noticed about myself is I started to have a lack of focus. I feel lost. My mood changes frequently. Everything goes wrong. I don’t know. I have no idea. It (HIV) ruined everything. | When I was diagnosed with HIV, I tried to attend, actually I forced myself to consistently attend the counselling sessions. Yes, I admit that the counsellor guided me, advised me to be strong when facing reality. I tried, but nothing works. Nothing has changed. I only see a blank future. | Every time I attended a session or treatment, I felt down. This does not mean I don’t like people, but I don’t know why I can’t bring myself to talk or discuss much with others. Even now, I just follow the procedures. I try to cooperate, but HIV is really making me think too much. It makes me sick, not only physically but mentally. I am very sure no one will accept this reality. |
| Participant 3 | With HIV, just assume you have lost everything in life, happiness and pleasure. Nothing more is left. I’m not just uncomfortable with the treatment, due to the side effects and needing to take a lot of medicine. The most uncomfortable thing is the need to engage with a certain commitment, like the need to attend regular counselling sessions. For me, it changes nothing. | I understand that counselling is intended to help and motivate people, but this is HIV, and I still cannot understand why they ask me to attend the counselling sessions. There is nothing for me. There is no one, and nothing can change this. It is HIV and I know that my life will end soon. | I am thinking too much by living with this disease and I know there is nothing that can be changed or hoped for. I just follow the treatment procedures. If I am not in the mood for it, I skip the appointment. | Honestly, it is not easy for me to continue life with HIV, and I believe there is no one who will accept and understand me now. With HIV, I need more space for myself. I prefer to share with those that I am close to, but not in the counselling sessions. I know that the counsellor tried to help me, but in terms of dealing with personal matters, I could not do this in the counselling sessions. Sometimes, I’m more comfortable talking with a medical doctor. |
| Participant 4 | Since the diagnosis, I am always thinking about the fact that I am dying. It makes me feel bad and that is why I feel uncomfortable about doing much in the treatment process. I thought I just needed to come to the hospital and take the medicine. The intervention sometimes made me feel a bit uncomfortable because it could be a long session, just to check on my progress. | I started to lose my spirit to continue my life. I spent a lot of money for medical, treatment, but there is still no change. I started feeling like giving up. I don’t even know how much longer I can survive. | HIV has completely taken over everything in my life. I have lost my happiness, my family, friends and career. I still have a job, but I know it can end at any time with my deteriorating health, painful medication and the struggles that I need to face outside. I have attended counselling sessions, but there is no hope for me. I am totally broken.  HIV has changed my life. I only feel like giving up on everything. It is too late now. I have no future living with this disease. I started to have no motivation to work and do the things I used to do. I will never go back to the way I was. | Since the diagnosis, I am always avoiding people. In treatment I am asked to attend a session with nurses or sometimes, the doctors. I just discuss with them the things related to my treatment. I have a feeling of assuming people will hurt me. I think I am too negative sometimes. |
| Participant 5 | I realize that most of the time my mood doesn’t allow me to do work or activities. I frequently feel uncomfortable with the environment and others. Yes, I admit that I skipped the treatment because of that feeling. It is not easy to deal with the treatment. | I felt nothing in the counselling session. This does not mean that I do not like the counsellor, but I think that they could not help much. Yes, I asked about and discussed a lot of things related to medicines and treatments, but the feeling that I have inside remains the same. I am always thinking of death and there are still many things I want to achieve in my life. | I always hate to think about the disease and treatment. I need to take a lot of medicines, but my condition remains the same. Most of the time I do think that it is useless to attend the treatment. But I still need to attend. | The thing is, I will be more comfortable staying alone and not talking much about this disease. I am scared that people will not easily accept me for who I am now. |
