# Supplementary material for: Counselling experiences among men having sex with men and living with HIV in Malaysia
Source: PLoS One. 2022 Sep 15;17(9):e0274251. doi: 10.1371/journal.pone.0274251 (PMC9477305; doi:10.1371/journal.pone.0274251)
Supplement: S1 File — (DOCX) [file pone.0274251.s002.docx]

Feedback to the Plos One Reviewer:

2. Interview guide:

| **Interview Number** | **Interview Questions** | |
| --- | --- | --- |
|  | **Bahasa Malaysia** | **English** |
| First Interview | 1. Boleh ceritakan tentang latarbelakang anda? 2. Bagaimana keadaan diri anda sekarang? 3. Bagaimana perasaan apabila disahkan menghidap HIV positif? 4. Bagaimana keadaan kehidupan anda selepas disahkan menghidap HIV? 5. Bagaimana anda melihat diri anda selepas disahkan menghidap HIV? | 1. How would you describe your background? 2. How would you describe your current situation? 3. What kind of feelings have you experienced since you became aware that you are HIV positive? 4. Tell me about your life after you discovered you are HIV positive. 5. How did you define yourself after you discovered you are HIV positive? |
| Second Interview | 1. Apa perubahan diri anda selepas disahkan HIV positif? 2. Boleh anda ceritakan pengalaman perubahan yang dihadapi selepas disahkan HIV positif? 3. Apa perasaan anda tentang perubahan yang dihadapi? 4. Apa yang boleh anda ceritakan tentang perubahan yang dihadapi selepas disahkan HIV positif? 5. Apa perasaan anda menghadapi cabaran selepas disahkan HIV positif? 6. Bagaimana anda melihat diri anda dalam menghadapi cabaran berkenaan? | 1. What changes have you faced since you discovered you are HIV positive? 2. Tell me about your experiences in facing the changes after you discovered you are HIV positive. 3. What are your feelings about facing the changes? 4. What can you tell me about the challenges you have faced since you discovered you are HIV positive? 5. What are your feelings about facing the challenges after you discovered you are HIV positive? 6. How do you see yourself facing the challenges? |
| Third Interview | 1. Boleh anda ceritakan tentang jangkaan anda terhadap kehidupan selepas dijangkiti HIV? 2. Apa pandangan anda tentang kehidupan selepas disahkan HIV positif? 3. Apa aktiviti yang anda sering lakukan selepas disahkan menghidap HIV positif? 4. Apa minat anda dalam kehidupan? 5. Apa perasaan and selepas melalui rawatan dan sesi kaunseling? 6. Apa kekuatan diri anda? | 1. Tell me about your expectations about your life after being infected with HIV. 2. What were your ideas about your life since you discovered you are HIV positive? 3. What activities did you do since you discovered you are HIV positive? 4. What are your interests in life? 5. What were your feelings after having been through the treatment and counselling session? 6. What are your strengths? |

3. Informed Consent attached: Item no. 7, 13 and 17

4. Participants’ details

a) the names of the hospitals patients were from: Hospital Sultanah Nur Zahirah (HSNZ) and Hospital Sungai Buloh (HSB)

| **Participant** | **Hospital** |
| --- | --- |
| 1 | HSNZ |
| 2 | HSB |
| 3 | HSB |
| 4 | HSNZ |
| 5 | HSNZ |

b) a description of any inclusion/exclusion criteria that were applied to participant recruitment:

Done, line 110 to 112

c) a statement as to whether your sample can be considered representative of a larger population, and

d) a description of how participants were recruited.

Done, line 114 to 120

5. In your Data Availability statement, you have not specified where the minimal data set underlying the results described in your manuscript can be found. PLOS defines a study's minimal data set as the underlying data used to reach the conclusions drawn in the manuscript and any additional data required to replicate the reported study findings in their entirety. All PLOS journals require that the minimal data set be made fully available. For more information about our data policy, please see <http://journals.plos.org/plosone/s/data-availability>:

Reviewer #1: Counselling experiences among HIV men who have sex with men in Malaysia
This is a paper that attempts to describe the experiences of men who have sex with men living with HIV. Overall, the manuscript is scanty and requires details to understand the context of counselling sessions. The number of participants in this study limits the interpretation of the results and the conclusion. Had the background on the study participants been provided, then likely would have explain the small sample. A description of how the authors were able to achieve saturation in this study is not provided neither is this cited as a limitation.

The overall paper would benefit from copy-editing.
Title: The title is ok, however reference should be made to men who have sex with men living with HIV and not HIV MSM.

Abstract: Introduction section is not clear what the problem is. Is increasing HIV incidence among MSMs associate with their experiences with counselling services? Methods section needs rewording;

Done.

Introduction: The section has several bold claims that are not substantiated. Line 50 has no reference.

Done

The sentence 51-54 referencing a WHO STI fact sheet (<https://www.who.int/news-room/fact-sheets/detail/sexually-transmitted-infections-(stis)>) does not seem to have relevant info on MSMs neither does it provide the estimates in European countries. It is unclear the % provided are increasing from what or when?

Done, Line 54 to 58

Line 61 –Not clear what “…adherence to HIV prevention programmes among HIV MSM was less than 70% dropping to 37.4% in 2017” means

Done, Line 66

Line 66 – Reference at the end (32) either is incorrect or the whole references in the document is a miss.

Done, Line 71 to 72

Difficult to follow what the problem is and what this manuscript is contributing to the problem. In general, the introduction needs to be revised. The authors should provide a background and the problem that this paper is responding to. What is the prevalence? Incidence? of HIV in Malaysia? What is the driver of the HIV incidence? What HIV counselling guidelines are available in Malaysia. Which of these is this manuscript focusing on, and why is it important?

Methods: The Methods section is mixed with the results (participants’ characteristics). The selection of the hospital is so vague – “these hospitals were selected because they have certain PLWH who were reported to regularly attend and engage….”. How were participants approached and selected to participate?

Done, Line 111 to 121

Was researcher (TN) part of the counsellors providing counselling services in any of the two hospitals? Some of the interview question are so ambiguous? Could this be due to direct translation from Bahasa Malaysia to English? For example; “How would you describe your current situation?” In what context?

Results: There are claims in the results that are not supported by the extracts – line 155 “the stories of the HIV MSM……that they were not ready to accept the status of their HIV”. This claim is carried through the findings. Another claim made and not supported by the extract is in line 159-160 “This is due to the poor trust towards self and others, affecting their motivation to live as HIV MSM”. The effect on motivation to live with HIV is not substantiated by the information provided. Trust for others seemed to be there for example in line 230-234, the participant shares their preference to share information to people close to them or their medical doctor.
The theme on hopelessness is not supported by any of the extracts – most if not all denotes trust issues.
This study would have benefited from interviewing counsellors to understand from their perspectives the processes of counselling and corroborate the information with those of the MSMs.

Discussion and conclusion: This section requires an overhaul should this manuscript be considered for publication. The basis of the discussion is skewed as it is not supported by the findings. The claim that the participants have feelings of emptiness and hopelessness in the counselling sessions because of their inability to accept their HIV status seems to be farfetched.
In summary, while this study has important findings that could inform how counselling should be tailored to improve care and positive living among MSMs, the manuscript falls short of presenting and arguing this case. It reads as thought the authors already had in mind what they wanted to share and were looking for data that can support their claim instead of the other way around.
