# Supplementary material for: Counselling experiences among men having sex with men and living with HIV in Malaysia
Source: PLoS One. 2022 Sep 15;17(9):e0274251. doi: 10.1371/journal.pone.0274251 (PMC9477305; doi:10.1371/journal.pone.0274251)
Supplement: Attachment — Submitted filename: Response to reviewers.docx [file pone.0274251.s003.docx]

Response to reviewers

|  | Reviewers’ feeback | Response to reviewers |
| --- | --- | --- |
| 1 | Please ensure that your manuscript meets PLOS ONE's style requirements, including those for file naming. The PLOS ONE style templates can be found at | Done |
| 2 | Please include a copy of the interview guide used in the study, in both the original language and English, as Supporting Information, or include a citation if it has been published previously. | Attached |
| 3 | Thank you for stating in the text of your manuscript "Written informed consent was obtained from the participants, and they were free from threats, injustice or manipulation". Please also add this information to your ethics statement in the online submission form. | Done |
| 4 | the names of the hospitals patients were from, | The names of the hospital cannot mention in the manuscript due to confidentiality.  These two are public hospital (fully funded by the government) and located at the peninsular of Malaysia. |
| 5 | a description of any inclusion/exclusion criteria that were applied to participant recruitment, | Done, page 5, line 73-78 |
| 6 | a statement as to whether your sample can be considered representative of a larger population, and | This sample cannot consider as representative of a larger population. |
| 7 | a description of how participants were recruited. | Done, page 5, line 80-82 |
| 8 | Counselling experiences among HIV men who have sex with men in Malaysia This is a paper that attempts to describe the experiences of men who have sex with men living with HIV. Overall, the manuscript is scanty and requires details to understand the context of counseling sessions. The number of participants in this study limits the interpretation of the results and the conclusion. Had the background on the study participants been provided, then likely would have explain the small sample. A description of how the authors were able to achieve saturation in this study is not provided neither is this cited as a limitation. | Rewrite has been done and it marked in blue.  This is a qualitative study with a grounded-theory approach and saturation has been mentioned (page 6, line 95-96) |
| 9 | The overall paper would benefit from copy-editing. | Done |
| 10 | The title is ok, however reference should be made to men who have sex with men living with HIV and not HIV MSM. | Correction has been done and marked in blue |
| 11 | Abstract: Introduction section is not clear what the problem is. Is increasing HIV incidence among MSMs associate with their experiences with counseling services? Methods section needs rewording; | Correction has been done and marked in blue |
| 12 | Introduction: The section has several bold claims that are not substantiated. Line 50 has no reference. The sentence 51-54 referencing a WHO STI fact sheet (<https://www.who.int/news-room/fact-sheets/detail/sexually-transmitted-infections-(stis)>) does not seem to have relevant info on MSMs neither does it provide the estimates in European countries. It is unclear the % provided are increasing from what or when? | Correction has been done and marked in blue |
| 13 | Line 61 –Not clear what “…adherence to HIV prevention programmes among HIV MSM was less than 70% dropping to 37.4% in 2017” means | Rewrite of this statement has been done and marked in blue |
| 14 | Line 66 – Reference at the end (32) either is incorrect or the whole references in the document is a miss. | Correction has been done and marked in blue |
| 15 | Difficult to follow what the problem is and what this manuscript is contributing to the problem. In general the introduction needs to be revised. The authors should provide a background and the problem that this paper is responding to. What is the prevalence? Incidence? of HIV in Malaysia? What is the driver of the HIV incidence? What HIV counseling guidelines are available in Malaysia. Which of these is this manuscript focusing on, and why is it important? | Rewrite has been done and marked in blue |
| 16 | Methods: The Methods section is mixed with the results (participants’ characteristics). The selection of the hospital is so vague – “these hospitals were selected because they have certain PLWH who were reported to regularly attend and engage….”. How were participants approached and selected to participate? Was researcher (TN) part of the counsellors providing counseling services in any of the two hospitals? Some of the interview question are so ambiguous? Could this be due to direct translation from Bahasa Malaysia to English? For example; “How would you describe your current situation?” In what context? | Rewrite has been done and marked in blue.  A copy of interview questions, in English and Bahasa Malaysia is attached. |
| 17 | Results: There are claims in the results that are not supported by the extracts – line 155 “the stories of the HIV MSM……that they were not ready to accept the status of their HIV”. This claim is carried through the findings. Another claim made and not supported by the extract is in line 159-160 “This is due to the poor trust towards self and others, affecting their motivation to live as HIV MSM”. The effect on motivation to live with HIV is not substantiated by the information provided. Trust for others seemed to be there for example in line 230-234, the participant shares their preference to share information to people close to them or their medical doctor. The theme on hopelessness is not supported by any of the extracts – most if not all denotes trust issues. | Rewrite has been done and marked in blue. |
| 18 | This study would have benefited from interviewing counselors to understand from their perspectives the processes of counseling and corroborate the information with those of the MSMs. | Thank you for the suggestion. Please refer to reference no 13. |
| 19 | Discussion and conclusion: This section requires an overhaul should this manuscript be considered for publication. The basis of the discussion is skewed as it is not supported by the findings. The claim that the participants have feelings of emptiness and hopelessness in the counseling sessions because of their inability to accept their HIV status seems to be farfetched | Rewrite has been done and marked in blue. |
| 20 | In summary, while this study has important findings that could inform how counseling should be tailored to improve care and positive living among MSMs, the manuscript falls short of presenting and arguing this case. It reads as thought the authors already had in mind what they wanted to share and were looking for data that can support their claim instead of the other way round. | Rewrite has been done and marked in blue. |
