# Supplementary material for: Counselling experiences among men having sex with men and living with HIV in Malaysia
Source: PLoS One. 2022 Sep 15;17(9):e0274251. doi: 10.1371/journal.pone.0274251 (PMC9477305; doi:10.1371/journal.pone.0274251)
Supplement: Attachment — Submitted filename: Response to reviewer.docx [file pone.0274251.s004.docx]

Response to reviewer

|  | Reviewer’ feeback | Response to reviewer |
| --- | --- | --- |
| 1 | The discussion section should include a paragraph on the strengths and weaknesses/limitation of this study. It is alluded to in the last sentence of the conclusion, although, I feel this is placed in the wrong section. | Correction has been done. New paragraph has been added and marked in blue, pg. 15, line 290-296 |
