# Supplementary material for: Counselling experiences among men having sex with men and living with HIV in Malaysia
Source: PLoS One. 2022 Sep 15;17(9):e0274251. doi: 10.1371/journal.pone.0274251 (PMC9477305; doi:10.1371/journal.pone.0274251)
Supplement: Attachment — Submitted filename: Additional comments.docx [file pone.0274251.s005.docx]

The authors have addressed the comments of the reviewers. I have only a few small things for them to add and then the manuscript can be officially accepted for publication.

I agree with the reviewer, the authors need to carefully read the manuscript and improve the language, including typos and etc.

Lines 35-36: “Factors contributing to the vulnerability of MSM living with HIV are related to stigma and hidden identity”

- Use the following reference to support it:

**Culture, social networks and HIV vulnerability among men who have sex with men in Indonesia. PLoS ONE. 2017;12(6):1-14.**

Lines 46-48: “MSM are widely exposed to behavioural risks of HIV infection because they have multiple sexual partners and have been found to consistently engage in casual sex without using condoms”.

- Use the following reference to support it:

**“Exploring determinants of unprotected sexual behaviours favouring HIV transmission among men who have sex with men in Yogyakarta, Indonesia. Global Journal of Health Science. 2017;9(8):47-56”.**
