# Supplementary material for: Counselling experiences among men having sex with men and living with HIV in Malaysia
Source: PLoS One. 2022 Sep 15;17(9):e0274251. doi: 10.1371/journal.pone.0274251 (PMC9477305; doi:10.1371/journal.pone.0274251)
Supplement: Attachment — Submitted filename: Response to reviewer.docx [file pone.0274251.s006.docx]

Response to reviewer

|  | Reviewer’ feeback | Response to reviewer |
| --- | --- | --- |
| 1 | Lines 35-36: “Factors contributing to the vulnerability of MSM living with HIV are related to stigma and hidden identity”  Use the following reference to support it:  **Culture, social networks and HIV vulnerability among men who have sex with men in Indonesia. PLoS ONE. 2017;12(6):1-14.**  Lines 46-48: “MSM are widely exposed to behavioural risks of HIV infection because they have multiple sexual partners and have been found to consistently engage in casual sex without using condoms”.  Use the following reference to support it:  **“Exploring determinants of unprotected sexual behaviours favouring HIV transmission among men who have sex with men in Yogyakarta, Indonesia. Global Journal of Health Science. 2017;9(8):47-56”.** | New references have been added, line 37 and line 50.  New references have been added at the refences section, reference no.3 and no.9. |
| 2 | 6. Reviewer #1: While a paragraph on strengths and limitations has been provided, this paragraph may require copy editing before publication is considered | Done. This section has been proof edited and the whole manuscript has been proof edited on 23/8/2022 |
